# Discovery of novel targets for multi-epitope vaccines: Screening of HIV-1 genomes using association rule mining

**DOI:** 10.1186/1742-4690-6-62

**Published:** 2009-07-06

**Authors:** Sinu Paul, Helen Piontkivska

**Affiliations:** 1 Department of Biological Sciences, Kent State University, Kent, Ohio 44242, USA

## Abstract

**Background:**

Studies have shown that in the genome of human immunodeficiency virus (HIV-1) regions responsible for interactions with the host's immune system, namely, cytotoxic T-lymphocyte (CTL) epitopes tend to cluster together in relatively conserved regions. On the other hand, "epitope-less" regions or regions with relatively low density of epitopes tend to be more variable. However, very little is known about relationships among epitopes from different genes, in other words, whether particular epitopes from different genes would occur together in the same viral genome. To identify CTL epitopes in different genes that co-occur in HIV genomes, association rule mining was used.

**Results:**

Using a set of 189 best-defined HIV-1 CTL/CD8+ epitopes from 9 different protein-coding genes, as described by Frahm, Linde & Brander (2007), we examined the complete genomic sequences of 62 reference HIV sequences (including 13 subtypes and sub-subtypes with approximately 4 representative sequences for each subtype or sub-subtype, and 18 circulating recombinant forms). The results showed that despite inclusion of recombinant sequences that would be expected to break-up associations of epitopes in different genes when two different genomes are recombined, there exist particular combinations of epitopes (epitope associations) that occur repeatedly across the world-wide population of HIV-1. For example, *Pol *epitope LFLDGIDKA is found to be significantly associated with epitopes GHQAAMQML and FLKEKGGL from *Gag *and *Nef*, respectively, and this association rule is observed even among circulating recombinant forms.

**Conclusion:**

We have identified CTL epitope combinations co-occurring in HIV-1 genomes including different subtypes and recombinant forms. Such co-occurrence has important implications for design of complex vaccines (multi-epitope vaccines) and/or drugs that would target multiple HIV-1 regions at once and, thus, may be expected to overcome challenges associated with viral escape.

## Background

In the course of viral infection, recognition of viral peptides by class I major histocompatibility complex (MHC) molecules and subsequent interactions of the peptide/MCH complex with the cytotoxic T lymphocytes (CTLs, or CD8+ T cells) plays an important role in the control of the infection [1,2]. Viral CTL epitopes (which are short viral peptides recognized by the immune system components, CTL and MHC class I molecules) are an integral – and critical – part of this recognition process, and amino acid changes at CTL epitopes have been shown to play a role in viral "escape" (in other words, evading recognition by the immune system) in human (HIV) and simian (SIV) immunodeficiency viruses [3-8]. In particular, in HIV certain CTL epitopes are subjected to consistent selective pressure from the host's immune system, leading to rapid accumulation of amino acid changes, while other CTL epitopes evolve under purifying selection pressure [9,10]. Furthermore, rapidly accumulating genetic diversity in the global HIV-1 pandemic [11] underlies a great need to develop vaccines that are protective against multiple subtypes and strains simultaneously.

The epitope-vaccine approach has been suggested as a strategy to circumvent the rapid rate of mutations in HIV-1 and the subsequent viral escape from the host's immune system as well as the development of resistance to anti-viral drugs [12-14]. The inclusion of CTL epitope sequences in vaccines has several advantages, including a possibility of targeting a majority of viral variants if highly conserved epitopes are used. Likewise, when epitopes from different genes or genomic regions are included in the same vaccine, such multi-epitope vaccines can induce broader cellular immune responses [15,16].

Several strategies can be used to develop multi-epitope vaccines, including (a) the generation of tetramer epitope vaccines with epitopes being chosen based on the presence of principal neutralizing determinant [12], (b) the generation of synthetic peptides with prediction of the candidate epitopes based on the peptide binding affinity of anchor residues *in silico*, focusing on those capable of binding to multiple HLA alleles [17], (c) the juxtaposition of multiple HLA-DR-restricted HTL epitopes [18] with epitope identification by screening of HIV-1 antigens for peptides that contain the HLA-DR-supertype binding motif [19]. However, inherent limitation of *in-silico *epitope predictions is generating a rather large number of initially predicted epitopes, many of which are false positives; and hence, there exists a need for subsequent experimental validation of many potential candidates [20-24]. Furthermore, because of the enormous genetic diversity of HIV, some predicted epitope candidates may be specific to only certain subtypes [21,25,26], whereas relying primarily on the extent of amino acid sequence conservation does not determine the potential immunogenicity [21]. Other methods, such as artificial neural networks [27] and hidden Markov models [28], also have limitations, such as adjustable values whose optimal values are hard to find initially, over fitting, overtraining and interpreting [29]. For example, in a study by Anderson et al. (2000) on experimental binding of 84 peptides to class I MHC molecules [30], there was no correlation between predicted versus experimental binding, and a high possibility of false-negatives. Thus, in this study we develop a novel strategy to identify best epitope candidates for multi-epitope vaccines from the pool of experimentally well-supported epitopes based on the association-rule mining technique.

Briefly, an association rule mining technique, which is a method that can detect association between items (frequent item sets) and formulate conditional implication rules among them [31-33], is used to examine relationships between 218 "best-defined" CTL epitopes (from the list of Frahm, Linde & Brander, 2007 [26]). Our results show that some CTL epitopes are significantly associated with each other so that they co-occur together in the majority of the reference viral genomes including circulating recombinant forms. At least 23 association rules were identified that involve CTL epitopes from 3 different genes, *Gag*, *Pol *and *Nef*, respectively. We also identified several combinations of 3 to 5 CTL epitopes that are frequently found together in the same viral genome despite high mutation and recombination rates found in HIV-1 genomes, and thus, can be used as likely candidates for multi-epitope vaccine development.

## Materials and methods

### HIV-1 genomic sequence data and alignment

Genomic nucleotide sequences of 9 protein-coding genes of HIV-1 were collected for 62 HIV-1 reference genomes from the 2005 subtype reference set of the HIV sequence database by Los Alamos National Laboratory (LANL) [34,35] (Table 1). These included 44 non-recombinant sequences from the groups M, N and O, and 18 circulating recombinant forms (CRFs). The M group was comprised of representatives of sub-subtypes A1, A2, F1 and F2, and subtypes B, C, D, G, H, J, K, respectively, of approximately 4 representative sequences from each category. This set of sequences was chosen since they allowed the diversity of each subtype to be roughly the same as for all available sequences in the database, similar to an effective population size. Moreover, they had full length genomes that covered all genes and major geographical regions (for criteria of selection of reference sequences, refer to [35]). Inclusion of CRFs allowed us to identify those highly conserved CTL epitopes that are shared between non-recombinant genomes and are also present in the majority of the recombinant reference genomes. Viral sequences were aligned at the nucleotide level as per amino acid alignment reconstructed with ClustalW, and were manually checked afterwards [36].

**Table 1:** List of 62 HIV-1 reference sequences (including 44 non-recombinant sequences, grouped by subtypes, and 18 circulating recombinant forms (CRFs) included in the study (2005 subtype reference set of the HIV sequence database, Los Alamos National Laboratory).

Subtype	Sequence name	Subtype	Sequence name
A1	A1.KE.94.Q23_17.AF004885	J	J.SE.93.SE7887.AF082394
	A1.SE.94.SE7253.AF069670		J.SE.94.SE7022.AF082395
	A1.UG.92.92UG037.U51190	K	K.CD.97.EQTB11C.AJ249235
	A1.UG.98.98UG57136.AF484509		K.CM.96.MP535.AJ249239
A2	A2.CD.97.97CDKTB48.AF286238	O	O.BE.87.ANT70.L20587
	A2.CY.94.94CY017_41.AF286237		O.CM.91.MVP5180.L20571
B	B.FR.83.HXB2-LAI-IIIB-BRU.K03455		O.CM.98.98CMU2901.AY169812
	B.NL.00.671_00T36.AY423387		O.SN.99.SEMP1300.AJ302647
	B.TH.90.BK132.AY173951	N	N.CM.02.DJO0131.AY532635
	B.US.98.1058_11.AY331295		N.CM.95.YBF30.AJ006022
C	C.BR.92.BR025-d.U52953		N.CM.97.YBF106.AJ271370
	C.ET.86.ETH2220.U46016		
	C.IN.95.95IN21068.AF067155	CRFs	01_AE.TH.90.CM240.U54771
	C.ZA.04.SK164B1.AY772699		02_AG.NG.-.IBNG.L39106
D	D.CD.83.ELI.K03454		03_AB.RU.97.KAL153_2.AF193276
	D.CM.01.01CM_4412HAL.AY371157		04_CPX.CY.94.CY032.AF049337
	D.TZ.01.A280.AY253311		05_DF.BE.-.VI1310.AF193253
	D.UG.94.94UG114.U88824		06_CPX.AU.96.BFP90.AF064699
F1	F1.BE.93.VI850.AF077336		07_BC.CN.97.CN54.AX149771
	F1.BR.93.93BR020_1.AF005494		08_BC.CN.97.97CNGX_6F.AY008715
	F1.FI.93.FIN9363.AF075703		09_CPX.GH.96.96GH2911.AY093605
	F1.FR.96.MP411.AJ249238		10_CD.TZ.96.96TZ_BF061.AF289548
F2	F2.CM.02.02CM_0016BBY.AY371158		11_CPX.GR.-.GR17.AF179368
	F2.CM.95.MP255.AJ249236		12_BF.AR.99.ARMA159.AF385936
	F2.CM.95.MP257.AJ249237		13_CPX.CM.96.1849.AF460972
	F2.CM.97.CM53657.AF377956		14_BG.ES.99.X397.AF423756
G	G.BE.96.DRCBL.AF084936		15_01B.TH.99.99TH_MU2079.AF516184
	G.KE.93.HH8793_12_1.AF061641		16_A2D.KR.97.97KR004.AF286239
	G.NG.92.92NG083.U88826		18_CPX.CM.97.CM53379.AF377959
	G.SE.93.SE6165.AF061642		19_CPX.CU.99.CU38.AY588970
H	H.BE.93.VI991.AF190127		
	H.BE.93.VI997.AF190128		
	H.CF.90.056.AF005496		

The last number in each sequence name is the GenBank accession number.

The summary of the average numbers of breakpoints in the CRF genomes was based on the breakpoint maps summarized at the HIV database at Los Alamos [37].

### CTL epitopes

The set of 218 CTL epitopes, described as "the best-defined HIV CTL epitopes" by Frahm, Linde & Brander (2007) [26] that included only those epitopes supported by strong experimental evidence in humans, was used. These epitopes, together with their respective genomic coordinates according to the reference HXB2 sequence (GenBank accession number K03455) [38], are described in Additional file 1.

### Selecting epitopes for association rule mining

**Figure 1:**
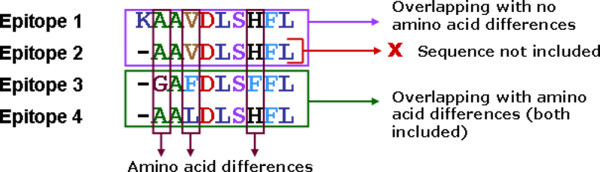
**Criteria for the inclusion of CTL epitopes**. The longer CTL epitope was selected from completely overlapping epitopes if they did not harbor any amino acid sequence differences among them, whereas both epitopes were included if at least one amino acid difference existed.

Of the 218 "best-defined" CTL epitopes, the subset of the most evolutionary conserved epitopes that are present across a majority of surveyed reference sequences was included according to the following criteria: (a) The epitope was present in at least one out of 62 reference sequences. (b) If two or more epitopes were completely overlapping with each other and there were no amino acid sequence differences, the longer epitope was selected. However, if overlapping epitopes harbored one or more amino acid difference from each other, all such epitopes were included (Figure 1). Even if two epitopes overlapped completely without any amino acid sequence differences within the overlap portion, it is possible that differences exist within the adjacent non-overlapping portions because of the difference in the epitope lengths. In such cases all epitopes were included. This step was taken to avoid generation of redundant association rules that are based on exactly the same amino acid sequences. Overall, 29 epitopes were removed from further analyses, resulting in a list of 189 epitopes that were included in the study (See Additional file 1 for details).

To determine whether the same associations exist among non-recombinant and circulating recombinant forms (CRFs), three data sets were created. The first sequence set (designated later as "62-all") included all 62 HIV-1 reference sequences used in the study, the second set included only 44 non-recombinant sequences ("44-non-CRFs") and the third set included 18 CRFs (designated as "18-CRFs"). Because of the requirement that an epitope be present as a "perfect match" in at least one sequence as described above, 1 and 29 epitopes were removed from the epitope lists for the second and third data sets, respectively. This resulted in lists of 188 and 160 epitopes, respectively (Additional file 1).

Additionally, one hundred "pseudo-datasets" of 62 sequences each (62 × 100) was created by randomly selecting sequences from the original sequence set (random sampling with replacement). Similarly to the bootstrap test widely used in phylogenetics [39], these pseudo-sets were used as controls to determine the significance of detected associations using the same threshold as the 62-all data set (i.e., 75% support and 95% confidence), in other words, whether identified associations in our original 62 sequence set could be attributed to the overrepresentation of certain sequence types by chance. The number of epitopes analyzed in each data set is given in Additional file 2. It should be noted that essentially the same association rules were identified in the pseudo-datasets as they were in the 62-all data set, which is consistent with the expectations that high values of support and confidence constraints used here already prune away most of the insignificant rules [32].

### Association rule mining

Association rule mining is a data mining technique that discovers relationships (associations, or rules) that exist within a data set [31-33,40]. One of the commonly known applications of association rule mining is "market basket" analysis [40-42]. However, in addition to marketing analysis, association rule mining has many useful applications to answer biological problems, including the discovery of relationships between genotypes and phenotypes in bacterial genomes [43], predicting drug resistance in HIV [44], and predicting MHC-peptide binding [45]. In this study, association rule mining was used to discover novel relationships between CTL epitopes that consistently co-occur together in viral genomes despite high mutation and recombination rates, so that such epitopes can be used as promising candidates in the design of multi-epitope vaccines.

Association rule mining was conducted using the Apriori algorithm [41] implemented in the program WEKA [40,46,47]. The initial minimum support was set at 0.75 and confidence at 0.95. The maximum number of rules identified was set at 5,000 for the 62-All and 44-non-CRFs data sets and at 50,000 for the 18-CRFs data set to ensure that all association rules above the support and confidence thresholds are captured. The support level was set rather high to include only associations among epitopes that were present in at least 75% of the reference sequences used. The confidence was set to 0.95 to generate only very strong associations, and all generated association rules were exhaustively enumerated and examined. Once identified, association rules were examined to identify "unique" rules, i.e., rules that combine associations between the same epitopes into a single, "unique" rule regardless of the order of epitopes within a rule (i.e., A occurs with B and B occurs with A are considered the same "unique" rule) (Table 2 and Additional file 3).

**Table 2:** Summary of the discovered CTL epitope association rules.

	**Data sets**			
**Association rules**	**62-all**	**44-non-CRFs**	18-CRFs *	**Pseudo-set**

Number of epitope associations with support >= 0.75 * & confidence >= 0.95	1961	1095	1867	1944

Unique epitope associations^#^				
Associations with 2 epitopes ^$^	46	48	45	46
Associations with 3 epitopes	217	166	71	217
Associations with 4 epitopes	153	102	59	151
Associations with 5 epitopes	59	26	27	58
Associations with 6 epitopes	9	2	7	9
Associations with 7 epitopes	0	0	1	0

***Total***	484	344	210	481

Unique epitope associations with epitopes from only one gene				
Epitopes from *Gag *only	9	12	3	9
Epitopes from *Pol *only	94	81	47	94
Epitopes from *Nef *only	0	0	0	0

***Total***	103	93	50	103

Unique epitope associations with epitopes from two genes				

*Gag-Pol*	329	234	145	326
*Pol-Nef*	26	11	7	26
*Nef-Gag*	3	1	1	3

***Total***	358	246	153	355

Unique epitope associations with epitopes from all three genes (*Gag-Pol-Nef*)	23	5	7	23

* Total number of associations includes all identified association rules that had a minimum support of 75% and 95% confidence. For CRFs, the support is 95%.

^# ^"Unique" rules combine associations between the same epitopes into a single, "unique" rule regardless of the order of epitopes within a rule (i.e., A occurs with B and B occurs with A are considered the same "unique" rule).

^$ ^i.e., association rules that involve two distinct CTL epitopes.

### Estimates of the nucleotide substitution rates

The relative degree of sequence divergence among reference sequences and different genomic regions was evaluated by comparing the number of synonymous and nonsynonymous substitutions. In particular, the number of synonymous nucleotide substitutions per synonymous site (dS) and the number of nonsynonymous nucleotide substitutions per nonsynonymous site (dN) were estimated by the Nei-Gojobori method [48] as implemented in the MEGA4 program [49]. This simple method was used because it is expected to have lower variance than more complicated substitution models [39]. The standard errors were estimated with 100 bootstrap replications. Pairwise dN and dS values were estimated for the so-called "associated" epitope regions (defined as epitopes that were found to be involved in any association rule), non-associated epitope regions (epitopes that were not involved in any association rule) and non-epitope regions (i.e., regions that did not harbor any CTL epitopes used in study), respectively.

## Results and discussion

### Mining for association rules

In order to identify CTL epitope regions that consistently co-occur together in the HIV-1 genomes, 189 CTL epitopes were mapped in 62 HIV-1 reference sequences (Table 1), where "perfect match" was recorded as "epitope presence", while one or more amino acid differences between the canonical CTL epitope sequence and respective HIV sequences were considered as "epitope absence", and association rule mining was applied to determine whether certain CTL epitopes consistently co-occurred together. Using the data mining tool WEKA [46,47], the initial minimum support and confidence values were set to 0.75 and 0.95, respectively, to ensure that we identified only the most frequently co-occurring epitopes. In other words, a minimum support value of 75% ensures that only epitopes that are present as a "perfect match" in at least 75% of the sequences are included in association rules (e.g., epitope A is present in at least 46 sequences out of 62). The support for the 18-CRFs data set was later raised to 0.95 (i.e., even more conservative) to limit the overall number of associations because this data set generated a lot more association rules with 75% support compared to the other data sets, as it had 31 CTL epitopes with at least 75% support whereas those for the 62-All and 44-non-CRFs data sets were 25 and 26, respectively. On the other hand, a level of confidence set to 95% indicates that the identified association rule (e.g., epitope A being associated with epitope B) will be present in at least 95% of the sequences where epitope A occurs. In the case of 62 reference sequences, that means at least 44 reference sequences had both epitopes present.

**Figure 2:**
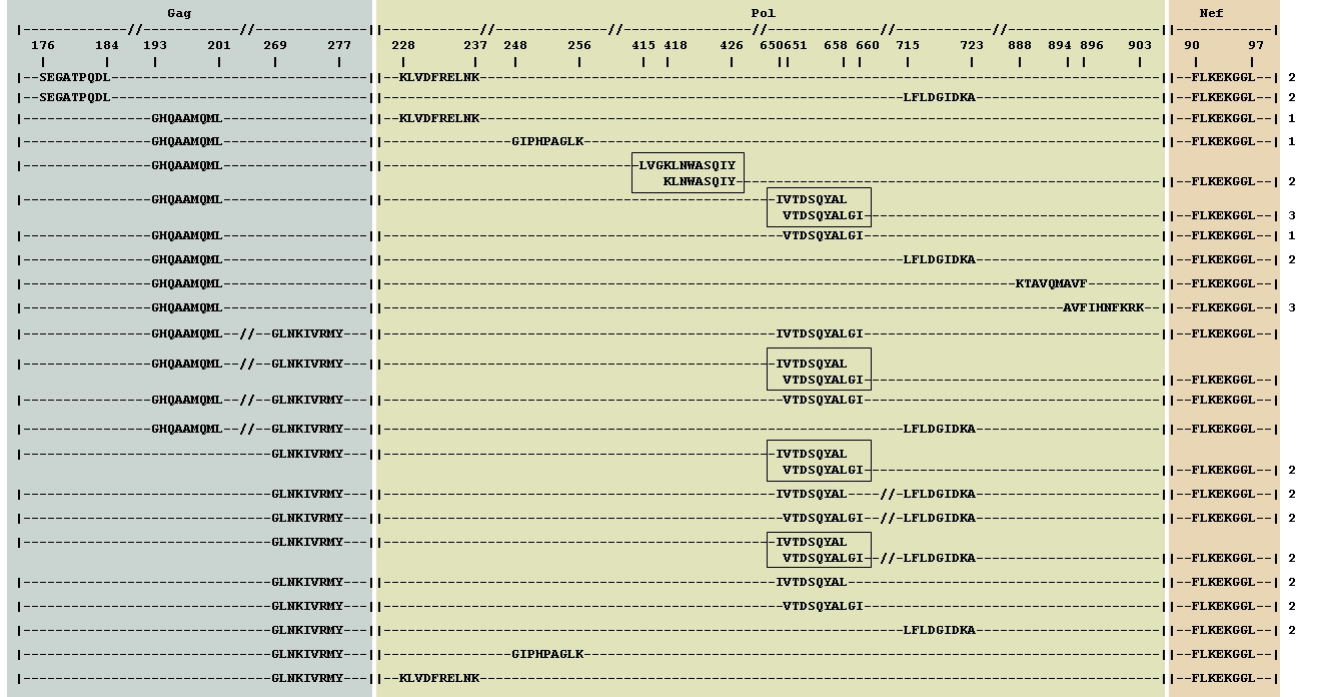
**Twenty-three association rules that include epitopes from three genes, and the respective amino acid sequences of the involved CTL epitopes (level of support >= 75%, confidence >= 95%), identified in 62 reference sequences of HIV-1 genomes (including 18 CRFs)**. Amino acid coordinates within each gene (*Gag*, *Pol *or *Nef*) are given relative to the epitope position in the HXB2 reference sequence (GenBank accession number K03455). Each line corresponds to a single association rule, and dashes designate amino acid sites that are NOT involved in the association rule. Drawn not to scale, "//" marks long stretches of non-included amino acid residues, and | indicates the border of a protein-coding gene. The numbers on the right side indicate the presence of the respective epitope association in other data sets: 1: 44-non-CRFs, 2: 18-CRFs and 3: Both 44-non-CRFs and 18-CRFs.

The results of the association rule mining are summarized in the Table 2. Initially, 1961 association rules were detected in the 62 sequences data set (1095 and 1867 for the 44-non-CRFs and 18-CRFs, respectively), of them 484, 344 and 210 were association rules involving unique combinations of epitopes (i.e., rules that A occurs with B and B occurs with A were considered the same "unique" rule), respectively. The majority of associations included 3 or 4 epitopes at a time; for example, the 62-all data set had 217 and 153 association rules involving 3 and 4 epitopes, respectively. However, a substantial amount of association rules was found to involve larger numbers of epitopes, 5 or 6 (Table 2). Among the unique epitope associations, a majority of them involved CTL epitopes harbored by the *Gag *and *Pol *genes for the 62-all sequence set (364 and 472 association rules included epitopes from the *Gag *and *Pol *genes, respectively), but only 52 association rules included an epitope from the *Nef *gene. Since *Gag *and *Pol *are located in adjacent genomic positions (and are somewhat overlapping), the physical proximity of the genes in the genome may be responsible for the existence of some association rules that involve epitopes from both of these genes (i.e., where recombination did not break up the association between epitopes). However, given the extremely high recombination rate in HIV-1, which was estimated to be as high as 2.8 crossovers per genome per replication cycle [50], epitopes from genes that are located far apart (such as *Pol *and *Nef*) would not be expected to be involved in many association rules, particularly those that occur with high support and confidence. Notably, our results identified at least 23 associations that involved epitopes located in 3 different genes, namely, *Gag*, *Pol *and *Nef *(shown in Figure 2). For example, epitopes *Gag *SEGATPQDL, *Pol *KLVDFRELNK and *Nef *FLKEKGGL were found to often co-occur in the same genome (Figure 2, **see also **Additional file 3). Notably, among the associated epitopes that are located on different genes, none was recognized by the same HLA allele within a genome or even by the alleles within the same supertype [51]. In the 3-epitope example above, these epitopes are recognized by the alleles HLA-B*4001, A*0301 and B*0801, which belong to the B44, A03 and B08 supertypes, respectively.

**Figure 3:**
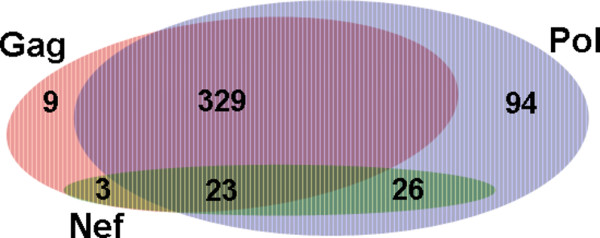
**Venn diagram showing the number of epitope association rules involving each gene**. Out of the 484 unique epitope associations, there were 9 associations in which epitopes from the *Gag *gene only (shown in red) were involved and 94 from the *Pol *gene only(blue). There was no association in which epitopes from solely *Nef *(green) were involved. There were 329 associations in which epitopes from *Gag *and *Pol *took part, whereas in 26 associations epitopes were only from the *Nef *and *Pol *genes, and in 3 associations epitopes were only from the *Gag *and *Nef *genes. There were 23 associations in which epitopes from all three genes were involved.

Overall, our results identified 358 association rules that involved epitopes from two different genes (mostly *Gag *and *Pol*) and 23 association rules that involve epitopes from three different genes (*Gag*, *Pol *and *Nef*). The Venn diagram shown on Figure 3 summarizes the distribution of different association rules among combinations of these three genes. As shown, the majority of all discovered unique association rules involved CTL epitopes from *Gag *and *Pol*, while among other categories of multi-gene association rules, majority involved combinations of epitopes from *Pol *and *Nef*. Similar results were obtained with the smaller 44-non-CRFs and 18-CRFs data sets, identifying 246 and 5 epitope associations from two and three genes, respectively, for the former data set, and 153 and 7 associations for the latter data set (Table 2, Additional file 3). Each of the epitope associations involving three genes were found to be present in more than 75% of the reference genomes, including all subtypes of the M group, N and O groups as well as the recombinant forms. When the N and O groups were excluded, the epitope associations were found to be present in more than 80% of the reference sequences. As an aside, the N and O groups are highly diverse viruses that represent only a small minority of HIV infections in West and Central Africa [52,53] and thought to originate in chimpanzee and gorilla zoonoses [54,55]. Presence of epitope association rules involving three genes is particularly notable for the18-CRFs data set, because it included a rather broad representation of circulating recombinant forms that are by definition products of recombination and often represent a complex mosaic of genomic pieces from multiple subtypes. On average, each recombinant subtype was inferred to have about 8 breakpoints (ranging from 2 to 16) across the entire genome, and included genomic segments of at least 2, and in some cases, 3 or more, distinct subtypes (as shown on the breakpoints maps available at the HIV database at Los Alamos). Furthermore, when the location of breakpoints and nature of rearrangements were considered, only 3 recombinant subtypes out of 18 used here had these three genes identified as originating from the same subtypes (i.e., CRF14_BG, CRF15_01B and CRF16_A2D). Yet, many of the associations found in larger data sets were also found in the 18-CRFs set, indicating that the amino acid segments that harbor these CTL epitopes are extremely conserved across a broad range of HIV-1 genomes.

Interestingly, one of the frequently associated epitopes found in three genes associations (Figure 2), *Nef *FLKEKGGL (HLA-B*08-restricted epitope) [56,8], also referred to as B8-FL8 epitope, is a known frequently targeted highly immunodominant epitope in HLA-B*08 individuals that often elicits a strong epitope-specific CD8+ T-cell response [57,58]. This epitope has also been shown to be targeted by specific T cell receptors that have unusually long complementarity determining regions 3 (CDR3) and capable of recognizing the escape mutants arising in that epitope, a response associated with slow disease progression [58]. Furthermore, the strong amino acid sequence conservation at this epitope region identified in our study is consistent with the clinical data that indicated a rather limited capacity of the virus to tolerate amino acid changes at that epitope, as evidenced by the lack of amino acid variation in some patients with persistent and strong CTL response despite being infected for over 13 years [8,58]. Overall, strong functional constraints on the virus and lower fitness of escape mutants are likely contributors to the high extent of sequence conservation of B8-FL8 epitope, and hence, it represents a promising vaccine candidate, although further studies are needed.

As Figure 2 shows, distribution of highly conserved epitope regions that participate in associations spanning three genes varied among and within genes. Notably, all 23 three-gene association rules included the same *Nef *epitope (B8-FL8 FLKEKGGL). The *Pol *gene had the highest number of associated epitopes (9) that differ from each other, while the *Gag *gene had 3 different epitopes involved in multiple association rules. Some of these associations included epitopes from the same adjacent/overlapping regions, e.g., *Gag *GLNKIVRMY is associated with the *Pol *IVTDSQYAL epitope and other adjacent/overlapping epitopes in at least 9 association rules (Figure 2). Other epitopes, such as *Gag *GHQAAMQML, instead participate in association rules that involved multiple non-overlapping epitope regions in the *Pol *gene. It is possible that different mechanisms are responsible for long-term evolutionary maintenance of different types of epitope associations, such as those that involve CTL epitopes from relatively closely located regions (within 200–300 codons apart), as well as associations that include epitopes from distantly located parts of the genome, although further studies are necessary. Overall, this approach allows us to identify co-evolving regions in viral genomes that are highly conserved at the amino acid level and are subjected to strong purifying selection eliminating the majority of amino acid changes that may occur in such regions.

### Selection at CTL epitopes involved in association rules

To assess the extent of evolutionary sequence conservation of the CTL epitopes that participated in the association rules, we compared the levels of nonsynonymous (amino acid altering) and synonymous substitutions in all pairwise comparisons of 62 HIV-1 genomes. The results are shown in Table 3, which lists average pairwise dN and dS values estimated for the epitope and non-epitope regions from the 62 HIV-1 reference genomic sequences. Here, the epitope regions are divided into two groups: (a) those epitopes that are involved in association rules and (b) those not involved. In all pairwise comparisons, the overall substitution trend is that the number of synonymous substitutions significantly exceeds that of nonsynonymous substitutions (i.e., dS >> dN, paired t test, p < 0.01). This indicates that purifying selection indeed plays a major role in the evolution of both the CTL epitopes and non-epitope regions, which is consistent with our previous results [9,10]. However, when the relative magnitude of nonsynonymous and synonymous changes was considered, epitopes that participated in association rules were found to have significantly lower dN values than either the other CTL epitopes or the non-epitope regions (ANOVA, p = 0.015), indicating that they are much more conserved at the amino acid but not at the nucleotide level. On the other hand, no significant differences were detected between dS values compared between these categories (p > 0.2). Similar results were obtained using nonparametric statistics (Kruskal-Wallis test, p = 0.002). These results indicate that purifying selection acting to preserve amino acid sequences of CTL epitopes is operating more strongly on the CTL epitopes that are found to be involved in association rules than on the non-associated epitopes, perhaps, due to stronger functional and structural constraints in these regions. However, further studies are necessary to determine the nature of these constraints.

**Table 3:** Average pairwise dN and dS values estimated at non-epitope and CTL epitope regions.

	dN	SE^#^	dS	SE	P value *
CTL epitopes involved in association rules	0.01696	0.00982	0.37794	0.20974	< 0.01
CTL epitopes not involved in association rules	0.12168	0.06814	0.50929	0.18780	< 0.01
Non-epitope regions	0.14698	0.10288	0.53472	0.12572	< 0.01

This involves all CTL epitopes and non-epitope regions from all the HIV-1 genomic sequences included in the study. CTL Epitope regions are divided into those involved in association rules and those not involved.

^# ^Standard errors were estimated with 100 bootstrap replications in MEGA4.

* In pairwise t-tests, the null hypothesis of dS = dN was rejected in all three comparisons.

### Significance of CTL epitope "participation" in the association rules

By design, our study was focused on the identification of highly supported association rules (support >= 75%), i.e., those that involve epitopes present in at least 75% of the sequences analyzed. Notably, not all CTL epitopes that are present in over 75% of sequences can be found in association rules (e.g., *Gag *CRAPRKKGC and *Pol *LVGPTPVNI while occurring in over 75% of the analyzed sequences, were not part of any association rule). As Table 4 shows, CTL epitopes from about 15 non-overlapping genomic regions participated in association rules; however, some genomic regions contributed more than one epitope (generally, these are overlapping epitopes). We also used a conservative level of confidence of 95% or higher, which can be interpreted as follows: if epitopes A and B are present together and are associated with epitope C with confidence of 0.95, we can conclude that whenever there are epitopes A and B in the same genome, epitope C will appear in the same genome with 95% probability or higher.

**Table 4:** Properties of 22 CTL epitopes that frequently co-occur together in the reference HIV-1 genomes (per the 62-all sequence set).

**Gene**	**Protein**	**Non overlapping genomic regions**	**Amino acid sequence**	HLA allele *	**Amino acid Coordinates**		**Number of "unique" association rules each epitope is involved**	**Number of association rules each region is involved**
							
					Start	End		
Gag	p24	1	SPRTLNAWV	B*0702	16	24	2	2
		2	SEGATPQDL^#^	B*4001	44	52	127	72
		3	GHQAAMQML^#^	B*1510, B*3901	61	69	214	110
		4	KRWIILGLNK^##^	B*2705	131	140	7	95
			GLNKIVRMY	B*1501	137	145	195	
			VRMYSPVSI	Cw18	142	150	1	

Pol	RT	5	IETVPVKL	B*4001	5	12	9	9
		6	KLVDFRELNK	A*0301	73	82	188	100
		7	GIPHPAGLK	A*0301	93	101	124	73
		8	TVLDVGDAY^###^	B*3501	107	115	38	31
		9	NETPGIRYQY	B18	137	146	14	9
			IRYQYNVL	B*1401	142	149	11	
		10	LVGKLNWASQIY	B*1501	260	271	112	51
			KLNWASQIY	A*3002	263	271	114	
	
	RT-RNase	11	IVTDSQYAL	Cw*0802	495	503	149	69
			VTDSQYALGI	B*1503	496	505	153	
	
	RT-Integrase	12	LFLDGIDKA	B81	560	8	121	68
	
	Integrase	13	KTAVQMAVF	B*5701	173	181	52	39
			AVFIHNFKRK	A*0301, A*1101	179	188	15	
			FKRKGGIGGY	B*1503	185	194	4	
		14	VPRRKAKII	B42	260	268	2	2

Nef		15	FLKEKGGL^###^	B*0801	90	97	52	34

These epitopes are harbored by 15 different protein-coding genomic regions. For each epitope and genomic region the number of "unique" association rules (as defined in Table 2) is shown, as well as the corresponding HLA alleles that recognize that particular CTL epitope.

* HLA alleles as defined in the HIV Molecular Immunology Database, Los Alamos National Laboratory.

^#^, ^## ^and ^### ^designate potentially promiscuous CTL epitopes that are: (^#^) recognized by alternative HLA alleles, (^##^) potentially embedded, or (^###^) shared between allele pairs (per [59]).

Overall, we were able to identify several highly conserved epitopes that are relatively widely spread across the worldwide HIV-1 population, and present not only in non-recombinant subtypes, but also in the circulating recombinant forms. Such highly conserved epitopes may be considered promising candidates for multi-epitope vaccine design, as they are likely to be targeted in a majority of HIV lineages, thereby increasing population coverage. However, in addition to being highly conserved, there are additional benefits in utilizing CTL epitopes identified as participants in association rules (such as those depicted on Figure 2). In particular, an association between epitopes generally implies that if one epitope from the rule is present in the viral genome, the other epitopes from the rule will also be present with high likelihood. Furthermore, because these epitopes may be located in different genes – and are often far apart from each other – a potential recombination – or a mutation – event may remove only some but not all target epitopes, and thus will only diminish the efficiency of a multi-epitope vaccine instead of completely disabling its action. Our earlier studies have identified at least 10 CTL epitope regions that exhibit evidence of persistent purifying selection (Piontkivska and Hughes 2004 [9], Table 2:  therein). Of these highly conserved epitopes, *Pol *epitope LFLDGIDKA (recognized by HLA-B81) is also found to be a part of several association rules identified in this study, including association rules spanning three genes and four CTL epitopes (in particular, 2 epitopes from *Gag *and 1 epitope from *Pol *and *Nef*, respectively), and as such, represents a promising candidate for multi-epitope vaccine development.

Because the HIV genomes and definitions of the CTL epitopes were drawn from the reference sequences and the list of "best-defined" epitopes of the HIV Sequence and HIV Immunology databases, respectively, neither patient's HLA haplotype, stage of infection nor CTL responses are known. However, some of the associated epitopes have been shown to be immunogenic in acute HIV-1 infection, particularly those participating in associations involving epitopes from three different genes, while some others have been shown to be strongly immunogenic in drug-naive patients (Additional file 4). Furthermore, while some CTL epitopes may certainly be prone to escape mutations when exposed to the immune pressure elicited by the restricting HLA allele, the associated epitopes identified in this study are recognized by different HLA alleles, with some combinations representing three different alleles from the same HLA locus. For example, epitope association of *Gag *SEGATPQDL, *Pol *LFLDGIDKA and *Nef *FLKEKGGL is recognized by the HLA-B*4001, B*81 and B*0801 alleles, respectively, and thus, it is unlikely to be recognized by all three alleles within the same patient. On the other hand, a recent study has shown that there is a promiscuity of some CTL epitopes where epitope presentation and CTL recognition can occur in the context of alternative, not restricting, HLA class I alleles, often from different HLA supertypes [59]. As shown in Table 4, five of the 22 associated epitopes have been designated as promiscuous [per [59]], with at least one promiscuous epitope identified in each gene (*Gag, Pol *and *Nef*). Therefore, inclusion of these epitopes may potentially enhance the efficiency of a multi-epitope vaccine across a broader range of host HLA haplotypes (although "functionally homozygous" individuals who express both original and alternative HLA alleles may be at disadvantage [55,60]). Further studies are needed to address the mechanisms of immune control of HIV infection through combinations of HLA alleles and CTL epitopes, particularly, promiscuous epitopes.

While our results demonstrated presence of several highly conserved – and identified to exist in association with each other – CTL epitopes in multiple HIV-1 reference genomes, including CRFs, the underlying functional significance of these regions for the virus remains poorly understood. Very few of the epitope regions found in association rules had such molecular features as glycosylstion, myristoylation, amidation, or phosphorylation sites. They also lacked any cell attachment motif or Leucine Zipper motif [61,62]. Yet, the highly conserved nature of these CTL epitopes hints at major functional significance of these regions. One possibility is that the strong sequence conservation is driven by functional constraints related to potential RNA secondary and tertiary structures formed by genomic regions of these epitopes, individually or in combination with each other. In such case it may be expected that the overall extent of sequence divergence will be lower at these epitopes than elsewhere in the genome, and indeed, both dN and dS values were found to be lower at the associated epitopes than at the other epitopes or non-epitope regions (Table 3).

It is also noteworthy that some epitopes are not involved in any association despite being present in more than 75% of the reference sequences, hinting at some underlying mechanism that holds the "associated epitopes" together. It is possible that the associated epitopes from different genes co-evolve together because of functional and structural constraints due to protein-protein interactions that are necessary for many viral processes [63]. Since some of the HIV proteins are expressed as polyproteins (such as Gag-Pol) [64], regulation of polypeptide processing in the cell is an important part of the viral life cycle and is often mediated by interactions between domains that belong to different processed proteins. For example, within Gag-Pol several regions that are located close to the N and C termini of protease (PR) have been shown to influence PR activation [65]. Likewise, modulating reverse transcriptase (RT) activation has been shown to have an effect on Gag-Pol interaction and polypeptide processing [66], while interactions between C terminal flexible loop of Nef and Gag-Pol polyprotein are essential for HIV assembly [67]. While molecular mechanisms of potential interactions involving associated epitope regions are currently unknown, these regions represent interesting candidates for future experimental studies to elucidate these interactions and their functional significance.

Our results revealed the presence of multiple associated co-evolving CTL epitope regions in HIV-1 genomes that are also significantly conserved across a broad range of HIV-1 subtypes and sub-subtypes. However, further studies are needed to ascertain the efficiency of these associated epitopes in multi-epitope vaccines as well as to uncover the underlying structural and/or functional constraints behind co-occurrences of the highly conserved epitopes.

## Conclusion

Application of association rule mining revealed that certain CTL epitope combinations (including epitopes from three different genes) consistently co-occur in HIV-1 genomic sequences present in major geographic regions around the world. Such epitopes that are both well supported by experimental evidence and highly conserved across different non-recombinant and recombinant forms of HIV-1 genomes can be considered as ideal candidates for multi-epitope vaccines against HIV-1.

## Abbreviations

HLA: Human Leukocyte Antigen; HTL: Helper T-Lymphocyte.

## Competing interests

The authors declare that they have no competing interests.

## Authors' contributions

SP did the analyses and wrote the manuscript. HP conceived and coordinated the study nd wrote the manuscript. All authors read and approved the final manuscript.

## Supplementary Material

Additional file 1 **Table S1 – Epitopes included in the study**. There were 218 epitopes in the "best defined CTL epitopes list" by Frahm et al. (2007). From this, 29 epitopes were removed from the 62-all data set because they did not satisfy the inclusion criteria. Additionally, one epitope from the 44-non-CRFs data set and 29 epitopes from the 18-CRFs data set were removed because of the inclusion criteria. Click here for file

Additional file 2 **Table S2 – Number of epitopes included in the study**. The number of epitopes included in the study as well as epitopes found in association rules in each gene. Click here for file

Additional file 3 **Table S3 – Unique epitope associations in the 62-all data set**. Each row represents one epitope association. Associated epitopes in each association are shown in adjacent columns. Presence of the association in other data sets is represented by 1 in respective columns. There are 484 unique associations in the 62-all data sets. Among them, 172 associations are present in the 44 – non-CRFs data set and 102 associations are present in the 18 – CRFs data set. The yellow color marks presence of the epitopes from all three genes in the 62-all data set. There are 23 associations of that kind. Click here for file

Additional file 4 **Table S4**. Stage of HIV-1 infection where the epitopes have been shown to be immunogenic, if known. Click here for file
